# Supplementary material for: Distress and Wellbeing among General Practitioners in 33 Countries during COVID-19: Results from the Cross-Sectional PRICOV-19 Study to Inform Health System Interventions
Source: Int J Environ Res Public Health. 2022 May 6;19(9):5675. doi: 10.3390/ijerph19095675 (PMC9101443; doi:10.3390/ijerph19095675)
Supplement: Supplementary file 1 [file ijerph-19-05675-s001.zip › ijerph-1671881-supplementary.pdf]

Table S1. Responses in each country for eWBI score, sampling method, and overall response rate.

	n	Study Invitation Sent to [10]:	Overall Response Rate [10]
Austria	110	Random national sample	28.0%
Belgium	406	Random national sample with additional convenience sample	29.7%
Bosnia and Herzegovina	26	Total population	5.5%
Bulgaria	76	Convenience national sample	94.3%
Croatia	97	Convenience national sample	11.7%
Czechia	91	Random national sample from four regions and from list of young practitioners	22.0%
Denmark	35	Total population	1.5%
Estonia	100	Total population	13.9%
Finland	59	Convenience national sample	15.5%
France	457	Total population	2.1%
Germany	228	Convenience sample in six areas	15.5%
Greece	52	Random national sample	94.0%
Hungary	165	Convenience national sample	23.4%
Iceland	23	Convenience national sample	23.8%
Ireland	154	Total population	12.2%
Israel	38	Convenience national sample	21.8%
Italy	101	Convenience national sample	25.6%
Kosovo*	34	Convenience sample in five areas	73.3%
Latvia	108	Total population	9.2%
Lithuania	38	Convenience national sample	22.5%
Malta	7	Total population	6.5%
Moldavia	44	Convenience sample from two municipalities	24.2%
Netherlands	141	Random national sample with additional convenience sample	18.9%
Norway	117	Total population	10.5%
Poland	124	Convenience national sample	10.4%
Portugal	172	Random national sample with additional convenience sample	22.9%
Romania	75	Convenience national sample	25.0%
Serbia	91	Convenience national sample	90.0%
Slovenia	132	Convenience national sample	19.8%
Spain	227	Convenience national sample	77.0%
Sweden	18	Convenience national sample	7.2%
Switzerland	73	Convenience sample	32.0%
Turkey	92	Convenience sample	27.9%
TOTAL	3711		27.8%

* All references to Kosovo, whether the territory, institutions, or population, in this project shall be understood in full compliance with the United Nations Security Council Resolution 1244 and the ICJ Opinion on the Kosovo declaration of independence, without prejudice to the status of Kosovo.

Table S2. Results of mixed effects logistic regression analysis of potential predictors for being at risk of distress in GPs (eWBI score ≥ 2) during the COVID-19 pandemic.

	**Mixed Effects Logistic Regression Models, Odds Ratio (95% CI) for eWBI Score ≥2**			
	**Model I: Odds Ratio (95% CI)**	**Model II: Odds Ratio (95% CI)**	**Model III: Odds Ratio (95% CI)**	**Model IV: Odds Ratio (95% CI)**
Intercept	1.87 (1.43, 2.46)***	1.11 (0.81, 1.52)	0.52 (0.32, 0.86)*	0.63 (0.33, 1.22)
GP experience				
30–39 years		Ref.	Ref.	Ref.
20–29 years		1.78 (1.44, 2.19)***	1.84 (1.47, 2.29)***	1.64 (1.27, 2.12)***
10–19 years		2.03 (1.63, 2.51)***	2.24 (1.79, 2.80)***	1.91 (1.46, 2.49)***
0–9 years		1.94 (1.57, 2.40)***	2.07 (1.65, 2.59)***	1.56 (1.20, 2.03)***
Number of GP staff in practice				
≥5			Ref.	Ref.
3–4			1.17 (0.92, 1.48)	1.09 (0.83, 1.44)
2			1.27 (0.98, 1.65)	1.25 (0.92, 1.70)
1			1.36 (1.06, 1.75)*	1.42 (1.06, 1.91)*
Practice location				
Big (inner)city			Ref.	Ref.
Suburbs			0.99 (0.75, 1.29)	0.97 (0.70, 1.33)
(Small) town			1.14 (0.91, 1.44)	1.20 (0.92, 1.57)
Mixed urban–rural			1.33 (1.06, 1.66)	1.25 (0.97, 1.62)
Rural			1.08 (0.86, 1.36)	1.25 (0.95, 1.64)
Patient population: chronic disease				
Below average			Ref.	Ref.
Approximately average			1.12 (0.79, 1.59)	1.06 (0.71, 1.59)
Above average			1.59 (1.11, 2.28)*	1.42 (0.94, 2.16)
Patient population: financial problems				
Below average			Ref.	Ref.
Approximately average			1.29 (1.07, 1.55)**	1.24 (1.00, 1.55)
Above average			1.46 (1.15, 1.84)**	1.40 (1.07, 1.84)*
Collaboration from neighborhood practices (0–4)				0.90 (0.83, 0.97)**
Adequate government support (0–4)				0.74 (0.68, 0.81)***
Responsibilities have increased (0–4)				1.31 (1.19, 1.44)***
Further training for amended responsibilities needed (0–4)				1.31 (1.20, 1.42)***
Enough protected time for reviewing guidelines/literature (0–4)				0.77 (0.72, 0.83)***
Intercept variance (s.e.)	0.58 (0.16)***	0.62 (0.17)***	0.60 (0.17)***	0.29 (0.10)**
MODEL INFORMATION				
Akaike's Information Criterion (AIC)	16482.19	16527.38	15642.09	13248.64
−2 Log Likelihood	16480.19	16525.38	15640.09	13246.64
Likelihood ratio test		45.19 (df=3)***	885.29 (df=11)***	2393.45 (df=16)***

* p<0.05; ** p<0.01; *** p<0.001.
